# The Epigenetic Role of miR-124 in HIV-1 Tat- and Cocaine-Mediated Microglial Activation

**DOI:** 10.3390/ijms232315017

**Published:** 2022-11-30

**Authors:** Palsamy Periyasamy, Annadurai Thangaraj, Muthukumar Kannan, Abiola Oladapo, Shilpa Buch

**Affiliations:** Department of Pharmacology and Experimental Neuroscience, University of Nebraska Medical Center, Omaha, NE 68198, USA

**Keywords:** cocaine, DNA methylation, epigenetics, HIV-1 associated neurocognitive disorders, HIV-1 Tat, microglia, neuroinflammation

## Abstract

HIV-1 and drug abuse have been indissolubly allied as entwined epidemics. It is well-known that drug abuse can hasten the progression of HIV-1 and its consequences, especially in the brain, causing neuroinflammation. This study reports the combined effects of HIV-1 Transactivator of Transcription (Tat) protein and cocaine on miR-124 promoter DNA methylation and its role in microglial activation and neuroinflammation. The exposure of mouse primary microglial cells to HIV-1 Tat (25 ng/mL) and/or cocaine (10 μM) resulted in the significantly decreased expression of primary (pri)-miR-124-1, pri-miR-124-2, and mature miR-124 with a concomitant upregulation in DNMT1 expression as well as global DNA methylation. Our bisulfite-converted genomic DNA sequencing also revealed significant promoter DNA methylation in the pri-miR-124-1 and pri-miR-124-2 in HIV-1 Tat- and cocaine-exposed mouse primary microglial cells. We also found the increased expression of proinflammatory cytokines such as IL1β, IL6 and TNF in the mouse primary microglia exposed to HIV-1 Tat and cocaine correlated with microglial activation. Overall, our findings demonstrate that the exposure of mouse primary microglia to both HIV-1 Tat and cocaine could result in intensified microglial activation via the promoter DNA hypermethylation of miR-124, leading to the exacerbated release of proinflammatory cytokines, ultimately culminating in neuroinflammation.

## 1. Introduction

The Centers for Disease Control and Prevention defines HIV-1 infection and drug abuse as intertwined epidemics, leading to compromised adherence to combined antiretroviral therapy and exacerbating HIV-1-associated neurocognitive disorders (HAND). Chronic low-level inflammation (mediated by viral proteins such as HIV-1 transactivator of transcription (Tat) and gp120, antiretrovirals, and abused drugs) has been considered a central driving element, as well as an essential correlate of HAND pathogenesis [1,2,3,4]. HIV-1 Tat is a neurotoxic, early viral protein consisting of 86–102 amino acids and is secreted from HIV-1-infected cells to control the viral replication [5,6,7]. HIV-1 Tat is also known to disturb cellular homeostasis via the augmented production of reactive oxygen species (ROS) that further change CNS functions [8,9,10,11]. Emerging studies also document the harmful effects of HIV-1 Tat protein in CNS cells, such as microglia, astrocytes, neurons, pericytes, oligodendrocytes, and endothelial cells, and the reported mechanisms include dysregulated autophagy, endoplasmic reticulum stress, defective lysosomal functions, dysfunctional mitochondria, inflammasome activation, and epigenetic modifications both in vitro and in vivo [12,13,14,15,16,17,18,19,20,21,22,23,24,25,26].

Similarly, the psychostimulant drug cocaine has also been reported to alter CNS functions in vitro and in vivo [27,28,29,30,31,32,33,34,35,36,37,38,39,40,41]. It is also well-known that HIV-1 Tat and cocaine cause neuronal dysfunction and are involved in HIV-associated neurocognitive disorder (HAND) [42,43,44,45,46,47,48]. People living with HIV-1 (PLWH) who abuse cocaine have exhibited the synergistic potentiation of viral replication and disease progression, in contrast with PLWH who do not use cocaine or HIV-1 negative cocaine users [49]. It is also well-known that neuroinflammation is an essential correlate of HAND pathogenesis in HIV-1-infected individuals [50]. Moreover, the increased production of proinflammatory cytokines by the activated microglia is also associated with cognition, memory and learning, and sensory functions in HAND patients with drug abuse [51,52,53,54]. Studies have also shown that HIV-1/HIV-1 Tat infection potentiates alterations in DNA methylation machinery, particularly the induction of DNMT1 expression and MeCP2/STAT3-mediated neuroinflammation [24,55]. Cocaine exposure in mice also elevates DNA methylation enzymes and MeCP2 binding, which decreases gene expression patterns in the nucleus accumbens [38]. Furthermore, cocaine exposure activates the mouse microglia via KLF4/TLR4 signaling axis both in vitro and in vivo [30]. These studies also signify the role of miR-124, a brain-enriched miRNA that plays a critical role in microglial quiescence and neuronal homeostasis [56,57,58]. In this study, we hypothesized that the exposure of mouse primary microglia to both HIV-1 Tat and cocaine could result in intensified microglial activation via the promoter DNA hypermethylation of miR-124, leading to the exacerbated release of proinflammatory cytokines, ultimately culminating in neuroinflammation.

## 2. Results

### 2.1. HIV-1 Tat and Cocaine Significantly Decreased the miR-124 Levels in Mouse Primary Microglial Cells

**Figure 1:**
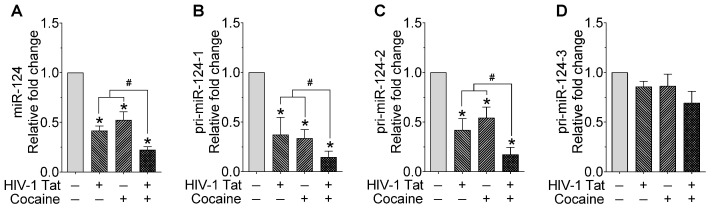
HIV-1 Tat and cocaine significantly decreased the miR-124 levels in mouse primary microglial cells. Representative qPCR analysis showing the expression of mature miR-124 (**A**), pri-miR-124-1 (**B**), pri-miR-124-2 (**C**), and pri-miR-124-3 (**D**) in HIV-1 Tat- (25 ng/mL) and cocaine (10 μM)-exposed mouse primary microglial cells for 24 h. Data are mean ± SEM from six independent experiments. Nonparametric Kruskal–Wallis one-way ANOVA followed by Dunn’s post hoc test was used to determine the statistical significance of multiple groups. * *p* < 0.05 versus control; ^#^ *p* < 0.05 versus HIV-1 Tat or cocaine.

To examine the combinatorial role of miR-124 in HIV-1 Tat- and cocaine-mediated microglial activation, we exposed mouse primary microglial cells (mPMs) to HIV-1 Tat (25 ng/mL) and cocaine (10 μM) for 24 h. After exposure, total RNA was isolated and used to determine the mature miR-124 expression using qPCR. As shown in Figure 1A, the expression of mature miR-124 was significantly decreased in mPMs exposed to HIV-1 Tat and cocaine compared with individual exposure of HIV-1 Tat and cocaine. Since three primary miR-124 (-1, -2, and -3) are responsible for the production of mature miR-124, we next determined the effects of HIV-1 Tat and/or cocaine on the expression levels of primary miR-124-1, -2, and -3 in mPMs by qPCR. As shown in Figure 1B,C, the expression of primary miR-124-1 and -2 was significantly downregulated in HIV-1 Tat- and cocaine-exposed mPMs compared with control cells and individual exposure. However, the expression of primary miR-124-3 was not altered following HIV-1 Tat and/or cocaine exposure to mPMs (Figure 1D). We also found that HIV-1 Tat- and/or cocaine-exposed mPMs showed increased cellular activation with ameboid morphology, thereby indicating the increased microglial activation (Figure S1A).

### 2.2. HIV-1 Tat and Cocaine Significantly Increased the Global Methylation and DNMT1 Levels in Primary Mouse Microglial Cells

**Figure 2:**
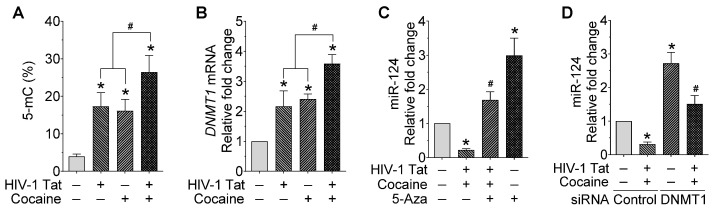
HIV-1 Tat and cocaine significantly increased the global methylation and DNMT1 levels in mouse primary microglial cells. (**A**) Quantification of 5-mC using ELISA in HIV-1 Tat (25 ng/mL) and cocaine (10 μM) exposed mouse primary microglial cells for 24 h. (**B**) Representative qPCR analysis showing the expression of DNMT1 mRNA in HIV-1 Tat (25 ng/mL) and cocaine (10 μM) exposed mouse primary microglial cells for 24 h. (**C**) Representative qPCR analysis showing the expression of miR-124 in mouse primary microglial cells pretreated with 5-Aza (5 μM) followed by HIV-1 Tat (25 ng/mL) and cocaine (10 μM) exposure for 24 h. (**D**) Representative qPCR analysis showing the expression of miR-124 in DNMT1 gene silenced mouse primary microglial cells exposed with HIV-1 Tat (25 ng/mL) and cocaine (10 μM) for 24 h. Data are mean ± SEM from six independent experiments. Nonparametric Kruskal–Wallis one-way ANOVA followed by Dunn’s post hoc test was used to determine the statistical significance of multiple groups. * *p* < 0.05 versus control; ^#^ *p* < 0.05 versus HIV-1 Tat or cocaine.

Since the exposure of mPMs to HIV-1 Tat and cocaine significantly decreased the expression of mature miR-124, as well as the primary miR-124-1 and -2, we next planned to find the global methylation status in mPMs following exposure to HIV-1 Tat and/or cocaine. Briefly, mPMs were exposed to HIV-1 Tat (25 ng/mL) and/or cocaine (10 μM) for 24 h. Following exposure, genomic DNA was isolated and used for quantifying global DNA methylation levels using 5-mC DNA ELISA Kit (Catalog No. D5325, Zymo Research, Orange, CA, USA), per the manufacturer’s instructions. As shown in Figure 2A, exposure of mPMs to HIV-1 Tat and/or cocaine significantly increased the global DNA methylation, compared with individual exposure. We next determined the effects of HIV-1 Tat and/or cocaine on the mRNA expression levels of DNMT1 in mPMs. mPMs were exposed to HIV-1 Tat (25 ng/mL) and/or cocaine (10 μM) for 24 h, following which total RNA was extracted to quantify the mRNA levels of the DNMT1 using qPCR. As shown in Figure 2B, the mRNA expression levels of DNMT1 in HIV-1 Tat- and/or cocaine-exposed mPMs are significantly than with individual exposure. Next, we wanted to find the specificity of DNMT1 in the HIV-1 Tat and cocaine-mediated downregulation of miR-124 in microglial cells. To confirm this, we pretreated the mPMs with 5-AZA (5 μM), a DNMT inhibitor, and checked the expression levels of miR-124 using qPCR. As shown in Figure 2C, 5-AZA pretreatment significantly abrogated the HIV-1 Tat- and/or cocaine-mediated downregulation of miR-124 in mPMs. We also determined the expression of miR-124 in DNMT1 silenced mPMs and found downregulation of miR-124 in HIV-1 Tat and/or cocaine-exposed, scrambled control transfected mPMs, which was significantly increased in DNMT1 silenced mPMs (Figure 2D). DNMT1 silencing efficiency was shown in Figure S1B. These results further confirmed the involvement of DNMT1 in HIV-1 and/or cocaine-mediated miR-124 downregulation.

### 2.3. HIV-1 Tat and Cocaine Significantly Increased the DNA Methylation Levels in Primary miR-124-1 and -2, in Microglia

Next, we isolated the genomic DNA from mPMs exposed to HIV-1 Tat and cocaine to determine the promoter DNA methylation levels of all three primary miR-124s. Briefly, mouse primary microglial cells were exposed to HIV-1 Tat (25 ng/mL) and/or cocaine (10 μM) for 24 h, followed by the extraction of genomic DNA for bisulfite-mediated nucleotide conversion. The PCR products were then sequenced to find the specific methylation of cytosine. We then determined the effects of HIV-1 Tat and cocaine on the DNA methylation levels of the primary miR-124-1 promoter in mouse primary microglial cells. Bioinformatics analyses showed dense CpG islands in the promoter region of primary miR-124-1. We thus designed two sets of primers so that their PCR products (fragments 1 and 2) encompassed approximately one kilobase of the promoter region.

**Figure 3:**
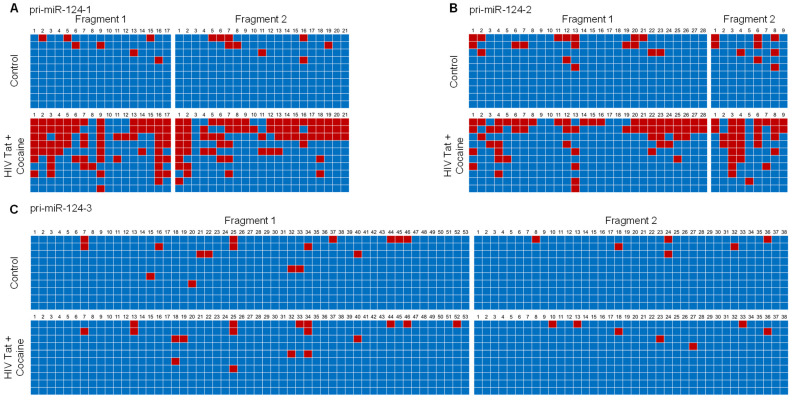
HIV-1 Tat and cocaine significantly increased the DNA methylation levels in primary miR-124-1 and -2, in microglia. Promoter DNA methylation of pri-miR-124-1 (**A**), prim-miR-124-2 (**B**), and pri-miR-1244-3 (**C**), using bisulfite-converted genomic DNA sequencing in HIV-1 Tat (25 ng/mL)- and cocaine (10 μM)-exposed mouse primary microglial cells. The promoter methylation status of sequenced data was analyzed using default threshold settings by BISMA software (http://services.ibc.uni-stuttgart.de/BDPC/BISMA, accessed on 25 July 2021).

As shown in Figure 3A, HIV-1 Tat and cocaine exposure of mPMs resulted in a substantial increase in promoter DNA methylation levels, both in fragment-1 as well as in fragment-2. Similar findings were observed in the promoter DNA methylation of primary miR-124-2 in HIV-1 Tat- and cocaine-exposed mPMs (Figure 3B). Our results demonstrated that HIV-1 Tat and cocaine significantly increased the methylation levels of primary miR-124-1 and -2 promoter regions. However, HIV-1 Tat and cocaine did not alter the CpG methylation rate in primary miR-124-3 promoter in mPMs (Figure 3C). Findings from these studies provided evidence that alterations in DNA methylation in primary miR-124 promoter are responsible for HIV-1 Tat- and cocaine-mediated miR-124 downregulation.

### 2.4. HIV-1 Tat and/or Cocaine Significantly Increased the Proinflammatory Cytokines in Microglia

**Figure 4:**
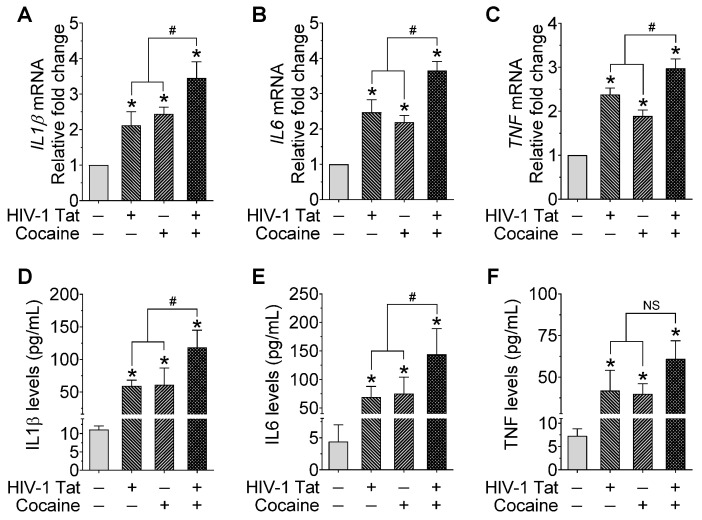
HIV-1 Tat and/or cocaine significantly increased the proinflammatory cytokines in microglia. Representative qPCR analysis showing the expression of IL1β (**A**), IL6 (**B**), and TNF (**C**) mRNA in HIV-1 Tat (25 ng/mL) and cocaine (10 μM) exposed mouse primary microglial cells for 24 h. Quantification of IL1β (**D**), IL6 (**E**), and TNF (**F**) levels using ELISA in HIV-1 Tat (25 ng/mL) and cocaine (10 μM) exposed mouse primary microglial cells for 24 h. Data are mean ± SEM from six independent experiments. Nonparametric Kruskal–Wallis one-way ANOVA followed by Dunn’s post hoc test was used to determine the statistical significance of multiple groups. * *p* < 0.05 versus control; ^#^ *p* < 0.05 versus HIV-1 Tat or cocaine; NS, Not significant.

Because miR-124 is involved in maintaining microglial quiescence, we next sought to investigate the effect of miR-124 on the HIV-1 Tat- and cocaine-mediated expression of proinflammatory cytokines in mPMs. Cells were treated with HIV-1 Tat and/or cocaine (as described above), followed by total RNA isolation and assessment for proinflammatory expression levels (IL1β, IL6, and TNF) mediators by qPCR and ELISA, respectively. As shown in Figure 4A–C, IL1β, IL6, and TNF expression levels significantly increased in mPMs exposed to HIV-1 Tat and/or cocaine compared with individual treatment. In addition, we performed ELISA to quantify these proinflammatory cytokines in the supernatant collected from mPMs exposed with HIV-1 Tat and/or cocaine for 24 h, and the results showed a significant increase in these proinflammatory cytokines in mPMs treated with HIV-1 Tat and/or cocaine (Figure 4D–F). Overall, these results demonstrated that the HIV-1 Tat- and cocaine-mediated activation of microglia involve the upstream downregulation of miR-124 through DNMT1-mediated miR-124 promoter DNA methylation.

### 2.5. HIV-1 Tat and/or Cocaine Significantly Increased Microglial Activation

**Figure 5:**
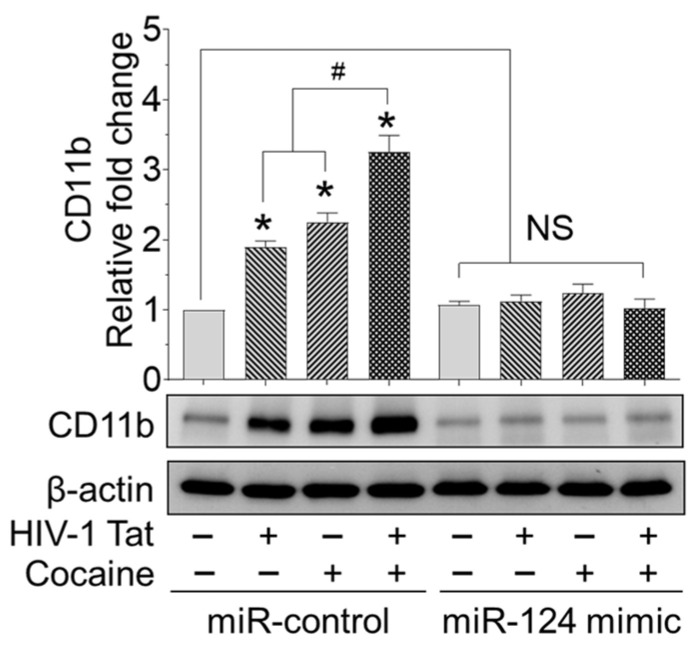
HIV-1 Tat and/or cocaine significantly increased microglial activation. Representative western blotting analysis showing the expression of CD11b in HIV-1 Tat (25 ng/mL) and cocaine (10 μM) exposed mouse primary microglial cells for 24 h. Data are mean ± SEM from six independent experiments. Nonparametric Kruskal–Wallis one-way ANOVA followed by Dunn’s post hoc test was used to determine the statistical significance of multiple groups. * *p* < 0.05 versus control; ^#^ *p* < 0.05 versus HIV-1 Tat or cocaine. NS: non-significant.

We further determined the effect of HIV and/or cocaine on microglial activation in mPMs transiently transfected with miRNA control and miR-124 mimic, followed by HIV-1 Tat and/or cocaine exposure for a period of 24 h. Then we isolated the proteins from these treated cells and performed western blotting for CD11b. As shown in Figure 5, we found a synergistic increase in microglial activation in mPMs transfected with miRNA control, followed by HIV-1 Tat and cocaine exposure, compared with individual exposure. Additionally, the HIV-1 Tat- and/or cocaine-mediated upregulated proinflammatory cytokines such as IL1β, IL6, and TNF were notably downregulated in DNMT1-silenced mPMs exposed to HIV-1 Tat and/or cocaine for 24 h (Figure S1C–E).

## 3. Discussion

This study demonstrates the epigenetic role of HIV-1 Tat and/or cocaine in the context of microglial activation with the involvement of promoter DNA methylation of miR-124. The rationale for choosing HIV-1 Tat dose is based on the fact that the concentration of HIV-1 Tat protein in the CSF is ~16 ng/mL, while that in the serum of HIV-1-infected individuals ranges from 0.1 to 40 ng/mL with actual concentration at tissue sites being even higher [59,60,61]. It has also been suggested that the local extracellular concentrations of HIV-1 Tat in the CNS could be even higher, especially in the locality of HIV-1-infected perivascular cells [62,63,64]. Therefore, similar concentrations of HIV-1 Tat were used in the present study. For cocaine dose, it has been revealed that the plasma concentrations of cocaine in humans, following intranasal cocaine administration, range between 0.4 and 1.6 µM [65], while the plasma cocaine concentrations in tolerant abusers reach levels up to 13 µM [66]. In addition, the cocaine concentrations in the postmortem brains of chronic cocaine users following acute intoxications have been reported to be higher than 100 µM [67]. We thus rationalized that 25 ng/ml HIV-1 Tat and 10 µM cocaine would be compatible with the average levels observed in HIV-1-infected people with cocaine abuse. The rationale for choosing time is based on our published literature showing the optimal downregulation of miR-124 at 24 h following HIV-1 Tat and cocaine exposure to mouse primary microglial cells [24,30,31].

Herein, we first determined the expression of mature miR-124 in mPMs exposed to HIV-1 Tat and cocaine and found the significant downregulation of mature miR-124, compared with individual exposure to HIV-1 Tat and cocaine. We also found that the decreased expression of mature miR-124 was due to the decreased expression of its primary miR-124-1 and -2 through promoter DNA hypermethylation. Further, the overexpression of miRNA in mPMs notably diminishes the augmented expression of proinflammatory cytokines and cellular activation. Thus, the modulation of the expression levels of miR-124 can be projected as a therapeutic target for attenuating the microglial activation mediated by HIV-1 Tat and/or cocaine.

Accumulating evidence confirmed the crucial role of miR-124 in microglial quiescence, and its decreased expression was correlated with microglial activation [56,57,58]. Additionally, microglial activation is positively linked with the proinflammatory cytokines storm with augmented production of reactive oxygen species, which is a confounding factor in the HAND pathogenesis [68]. In this study, we focused on the involvement of miR-124 in microglial activation following HIV-1 Tat and/or cocaine exposure. The justification for using HIV-1 Tat as a substitute for infection in cell culture is as follows. It is well recognized that despite cART, HIV-1 Tat remains present in the CNS and lymph nodes of treated patients [69,70]. There is also the indication of low-level virus replication in the CNS of cART-treated subjects [69,70], thereby providing rationality to the use of HIV-1 Tat as a surrogate of HIV-1 infection in cell culture studies.

Further, the decreased expression of miR-124 in HIV-1 Tat and/or cocaine-exposed mPMs revealed the reciprocal association between miR-124 and microglial activation. It is well known that dysregulated miRNA expression is often associated with altered DNA methylation of the respective miRNA encoding genes, thereby contributing to several disease processes [71]. In this study, we also showed that HIV-1 Tat and/or cocaine-exposed mPMs elicited elevated levels of 5-mC (an indicator of global DNA methylation) along with the increased expression of DNA methylation enzyme, such as DNMT1, thereby indicating a possible involvement of epigenetic DNA modifications in the regulation of miR-124. 

Further, the decreased expression of miR-124 in HIV-1 Tat and/or cocaine-exposed mPMs revealed the reciprocal association between miR-124 and microglial activation. It is well known that dysregulated miRNA expression is often associated with altered DNA methylation of the respective miRNA encoding genes, thereby contributing to several disease processes [71]. In this study, we also showed that HIV-1 Tat and/or cocaine-exposed mPMs elicited elevated levels of 5-mC (an indicator of global DNA methylation) along with the increased expression of DNA methylation enzymes, such as DNMT1, thereby indicating a possible involvement of epigenetic DNA modifications in the miR-124 regulation.

To further understand the detailed molecular mechanism(s) involving the role of downregulated miR-124 in microglial activation and the involvement of epigenetic DNA methylation, we performed bisulfite genomic DNA sequencing in HIV-1 Tat and cocaine-exposed mPMs and found increased CpG methylation in the promoter of primary miR-124-1 and -2 (but not in the primary miR-124-3). One likely reason for the lack of change in DNA methylation of the primary miR-124-3 promoter could be its part as a compensatory check that likely functions only in the absence of the other two miRs. Our findings are consistent with those by others demonstrating that both HIV-1 infection, as well as HIV-1 Tat and abused drugs, can induce the expression of DNMTs in lymphomas, thereby leading to increased genomic DNA methylation with dysregulated gene and miRNA expression [72,73,74]. Also, promoter DNA methylation of primary miR-124s has been reported in various disease conditions, including cancers, drug addiction, and HIV-1 infection [24,30,31,75,76,77,78,79,80]. 

Our earlier studies reported miR-124 downregulation in microglial cells exposed to HIV-1 Tat and cocaine (individual exposure) via promoter DNA methylation, which resulted in the activation of TLR4-mediated and MECP2-STAT3 signaling [24,30,31]. Other published studies have also investigated the role of miR-124 in CNS inflammation and age-related neurodegenerative diseases with a special focus on microglia and neurons [81,82,83,84]. The mechanism of miR-124 involvement in Alzheimer’s disease (AD) mainly interfered with the clearance of the amyloid precursor protein and demonstrated that there was a negative regulatory relationship between miR-124 and BACE1 expression and that miR-124 could be a promising therapeutic target in patients with AD [85,86]. In brain samples of AD patients and AD mouse model, the expression of miR-124 was downregulated and in turn, responsible for the post-transcriptional regulation of APP expression [86,87,88]. It is also known that miR-124 is one of the most significantly dysregulated miRs in temporal cortex samples from patients with AD, and it predicted that the dysregulation of miR-124 expression in the human brain might contribute to the pathogenic changes of AD [89]. In addition to AD, microglial miR-124-mediated chronic inflammatory response in the brain is reported in the onset and progression of Parkinson’s disease, with the involvement of increased cell apoptosis, dysregulated autophagy, 6-OHDA-induced neuronal injury, and NFKB signaling [90]. There are a few studies on the relationship between miR-124 and HD and ALS. It was reported that miR-124 was downregulated in the cortex and hippocampus of HD transgenic mice [91,92], and the neural stem cells from the ALS transgenic mice [93].

Drug addiction is now documented as a neuroinflammation-related disease, as several abused drugs modulate the levels of miR-124. It has been reported that the chronic administration of cocaine decreased miR-124 levels in the NAc, and that the downregulation of miR-124 was critical for cocaine-induced synaptic plasticity and behavioral changes [94]. These findings agree with our results that cocaine exposure leads to the downregulation of miR-124, ultimately culminating in microglial activation. The role of miR-124 in drug addiction has also been reported in overexpression studies, wherein lentivirus-miR-124 transduction in the NAc resulted in the attenuation of cocaine-induced CPP [95]. Interestingly, reduced levels of miR-124 have also been found in the ventral tegmental area of methamphetamine self-administering rats by microarray analysis [96]. Reports also demonstrate decreased miR-124 levels in ethanol-withdrawn rats, which was likely attributed to histone acetylation [97]. All these studies allude to the fact that miR-124 downregulation is critical for reward-related behavior changes induced by abused drugs, but detailed mechanisms remain unexplored. Overall, our findings are consistent with the published reports and lend further credence to the fact that the HIV-1 Tat- and cocaine-mediated downregulation of microglial miR-124 was involved in increased microglial activation mediated by the promoter DNA methylation of miR-124. It is also well-known that neuroinflammation is an essential correlate of HAND pathogenesis in HIV-1-infected individuals [50]. Moreover, the increased production of proinflammatory cytokines by the activated microglia is also associated with cognition, memory and learning, and sensory functions in HAND patients with drug abuse [51,52,53,54].

## 4. Materials and Methods

### 4.1. Mouse Primary Microglia

Mouse primary microglial cultures were prepared from one- to three-day-old newborn pups of either sex, bred from C57B1/6 under standard conditions, as published previously [98], with slight modifications [13,18,24]. The purity of the isolated microglia was assessed by immunocytochemistry using the antibody specific for Iba-1 (Catalog No. 019–19741, Wako Pure Chemical Industries, Ltd., Irvine, CA, USA; 1:200 dilution), and used if it is >95% pure.

### 4.2. TaqMan^®^ miRNA Assays

miR-124 expression was determined using TaqMan^®^ miRNA assays, as described previously [24,30,31]. Briefly, isolated total RNA was reverse-transcribed to synthesize cDNA for individual miRNA using specific miRNA primers (miR-124 Assay ID: 001182; U6 snRNA Assay ID: 4427975; Thermo Fisher Scientific, Waltham, MA, USA) for the following PCR reaction. Each PCR reaction was carried out in triplicate, and six independent experiments were run. TaqMan^®^ miRNA assays were performed using an Applied Biosystems^®^ QuantStudio™ 3 Real-Time PCR System (Applied Biosystems, Grand Island, NY, USA). The expression level of miR-124 was calculated by normalizing with U6 snRNA.

### 4.3. Global DNA Methylation

Genomic DNA extracted from mPMs exposed to HIV-1 Tat and/or cocaine were used to determine the global DNA methylation (5-methylcytosine) using 5-mC DNA ELISA Kit (Catalog No. D5325, Zymo Research, Orange, CA, USA), per the manufacturer’s instructions.

### 4.4. Bisulfite-Converted Genomic DNA Sequencing

Bisulfite-converted genomic DNA sequencing was performed using the protocol described previously, with minor changes [24,31]. Briefly, genomic DNA extracted from mouse primary microglial cells was exposed to bisulfite conversion by EZ DNA Methylation-Direct Kit (Catalog No. D5021, Zymo Research, Orange, CA, USA). The bisulfite-modified DNA was amplified by bisulfite sequencing PCR using Platinum PCR SuperMix High Fidelity (Catalog No. 12532016, Thermo Fisher Scientific, Waltham, MA, USA), with primers specific to mouse primary miR-124-1, primary miR-124-2, and primary miR-124-3 promoter regions. Subsequently, the amplified PCR products were purified by gel extraction with the Zymoclean Gel DNA recovery kit (Catalog No. D4008, Zymo Research, Orange, CA, USA), followed by cloning into pCR4-TOPO vectors using the TOPO TA Cloning kit (Catalog No. K457502, Thermo Fisher Scientific, Waltham, MA, USA). The recombinant plasmids were transformed into One Shot TOP10 chemically competent *Escherichia coli* (Catalog No. K459540, Thermo Fisher Scientific, Waltham, MA, USA) using the conventional chemical transformation method. Plasmid DNA was isolated from approximately ten independent clones of each amplicon with PureLink Quick Plasmid Miniprep Kit (Catalog No. K210011, Thermo Fisher Scientific, Waltham, MA, USA), then sequenced (High-Throughput DNA Sequencing and Genotyping Core Facility, University of Nebraska Medical Center, Omaha, NE, USA) to determine the status of CpG methylation. Only the clones with an insert containing greater than 99.5% bisulfite conversion (i.e., nonmethylated cytosine residues to thymine) were included in this study. The sequence data of each clone were analyzed for methylation in the miR-124 promoter by BISMA software (http://services.ibc.uni-stuttgart.de/BDPC/BISMA; accessed on 25 July 2021) using default threshold settings.

### 4.5. miR-124 Mimic Transfection

Mouse primary microglia were seeded into six-well plates (3 × 10^5^ cells per well) and were transiently transfected with 30 pmol of miR-124 mimic, and miRNA control using Lipofectamine™ RNAiMAX (Catalog No. 13778150, Thermo Fisher Scientific, Waltham, MA, USA), as described [24,30,31]. Following transfection, cells were exposed to HIV-1 Tat (50 ng/mL) and/or cocaine (1 μM) for another 24 h, and total RNA and proteins were extracted for further investigation, as indicated.

### 4.6. TaqMan^®^ miRNA Assays for miR-124

The expression of miR-124 was quantified using TaqMan^®^ miRNA assays, as described [24,30,31]. Briefly, total RNA was extracted using Quick-RNA™ MiniPrep Plus (Catalog No. R1058, Zymo Research, Orange, CA, USA), per the manufacturer’s protocol. Thus, the total RNA isolated was reverse transcribed to synthesize cDNA for individual miRNA using specific miRNA primers from the TaqMan^®^ miRNA assays and the TaqMan^®^ miRNA Reverse Transcription kit (Catalog No. 4366597, Thermo Fisher Scientific, Waltham, MA, USA). The reverse transcription product was then diluted 1:10 for the following PCR reaction. Each PCR reaction was carried out in triplicate, and six independent experiments were run. TaqMan^®^ miRNA assays were performed using an Applied Biosystems^®^ QuantStudio^™^ 3 Real-Time PCR System (Applied Biosystems, Grand Island, NY, USA). The expression level of miR-124 was calculated by normalizing with U6 snRNA.

### 4.7. Quantitative Polymerase Chain Reaction (qPCR)

qPCR experiments were performed according to our published protocol [18,24,30,31,36]. Briefly, total RNA was extracted using Quick-RNA™ MiniPrep Plus (Catalog No. R1058, Zymo Research, Orange, CA, USA), per the manufacturer’s protocol. Reverse transcription reactions were performed using iScript™ Reverse Transcription Supermix for RT-qPCR (Catalog No. 1708841, Bio-Rad, Hercules, CA, USA), per the manufacturer’s instructions. qPCRs were completed using TaqMan^®^ Universal PCR Master Mix, no AmpErase^®^ UNG (Catalog No. 4324018, Thermo Fisher Scientific, Waltham, MA, USA) in an Applied Biosystems^®^ QuantStudio™ 3 Real-Time PCR System (Applied Biosystems, Grand Island, NY, USA). Each reaction was carried out in triplicate, and six independent experiments were run. *Gapdh* was used as a housekeeping control for the normalization, and the fold change in expression was obtained by the 2^−ΔΔCT^ method.

### 4.8. Western Blotting

Western blotting was performed using standard procedures, as published previously [18,24,30,31,36]. Briefly, the control and treated microglial cells were harvested and lysed using the 200 μL of RIPA buffer (Catalog No. 9806, Cell Signaling Technology, Danvers, MA, USA). Lysates were centrifuged at 12,000× *g* for 10 min at 4 °C, and the protein content of the supernatant was determined by a BCA assay using Pierce^™^ BCA Protein Assay Kit (Catalog No. 23227, Thermo Fisher Scientific, Waltham, MA, USA), as per the manufacturer’s instructions. Equal amounts of soluble proteins were resolved in a 10% sodium dodecyl sulfate-polyacrylamide gel electrophoresis, followed by blotting onto a polyvinylidene fluoride membrane (Catalog No. IPVH00010, Millipore, Danvers, MA, USA). Then, the membranes were blocked with 5% nonfat dry milk (in 1× TTBS buffer) for one hour at room temperature, followed by overnight incubation with the indicated primary antibodies at 4 °C. After washing three times, membranes were incubated with a secondary antibody for one hour at room temperature. Next, the protein signals were visualized using Super Signal West Pico Chemiluminescent Substrate (Catalog No. 34078, Thermo Fisher Scientific, Waltham, MA, USA). Each band intensity was normalized to the internal control, β-actin (Catalog No. A5316, Sigma-Aldrich, St. Louis, MO, USA; 1:5000 dilution), and the data were presented as a relative fold change by using ImageJ analysis software [99].

### 4.9. ELISA

The supernatants from HIV-1 Tat and/or cocaine exposed to mPMs were used to determine the levels of proinflammatory cytokines, such as IL1β, IL6, and TNF, using ELISA, per the manufacturer’s instructions.

### 4.10. Statistical Analysis

All the data were expressed as mean ± SEM, and statistical significance was determined using GraphPad Prism version 6.01 (San Diego, CA, USA). The detailed statistical analysis used is shown in each figure caption for all studies. Non-parametric Kruskal–Wallis one-way ANOVA, followed by Dunn’s post hoc test, was used to find the statistical significance between multiple groups. Values were statistically significant when *p* < 0.05.

## 5. Conclusions

In conclusion, this study demonstrated that the HIV-1 Tat- and/or cocaine-mediated downregulation of miR-124 involved the DNA methylation of primary miR-124-1 and primary miR-124-2 promoters. Furthermore, downregulated miR-124 led in turn to the increased expression of proinflammatory cytokines and ensuing microglial activation.

## Data Availability

The data that support the findings of this study are available from the corresponding author upon reasonable request.

## Supplementary Materials

The following supporting information can be downloaded at: https://www.mdpi.com/article/10.3390/ijms232315017/s1.
