# Supplementary material for: The Epigenetic Role of miR-124 in HIV-1 Tat- and Cocaine-Mediated Microglial Activation
Source: Int J Mol Sci. 2022 Nov 30;23(23):15017. doi: 10.3390/ijms232315017 (PMC9738975; doi:10.3390/ijms232315017)
Supplement: Supplementary file 1 [file ijms-23-15017-s001.zip › ijms-2015355-supplementary.pdf]

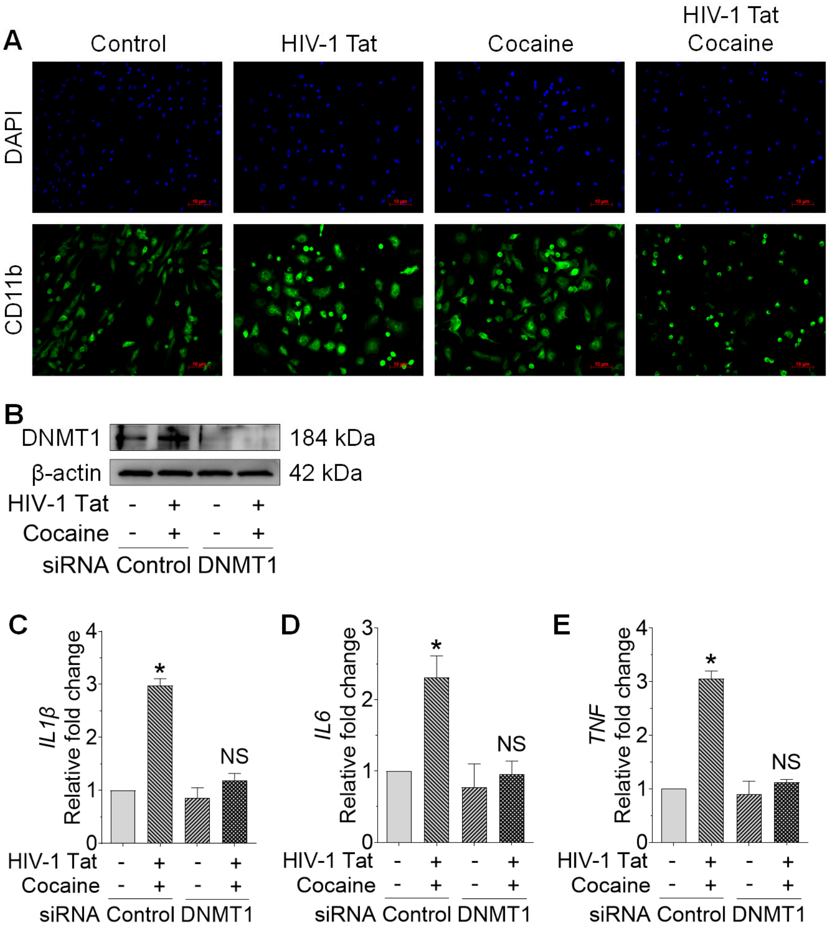

Figure S1. (**A**) Immunostaining showed the microglial morphology in HIV-1 Tat and/or cocaine-exposed mouse primary microglia. Scale bar: 10 μM. (**B**) Representative western blot image showed the silencing efficiency of DNMT1 in mouse primary microglia transfected with scrambled and DNMT1 siRNA followed by HIV-1 Tat and cocaine exposure for 24 hours. Representative qPCR analysis showing the expression of *IL1β* (**C**), *IL6* (**D**), and *TNF* (**E**) mRNA in mouse primary microglia transfected with scrambled and DNMT1 siRNA followed by HIV-1 Tat and cocaine exposure for 24 hours. Data are mean ± SEM from six independent experiments. Nonparametric Kruskal–Wallis one-way ANOVA followed by Dunn's post hoc test was used to determine the statistical significance of multiple groups. *$p < 0.05$ versus control; NS: Not significant.
